# Time-of-day influences resting-state functional cortical connectivity

**DOI:** 10.3389/fnins.2023.1192674

**Published:** 2023-05-31

**Authors:** Costanza Iester, Monica Biggio, Simone Cutini, Sabrina Brigadoi, Charalambos Papaxanthis, Giampaolo Brichetto, Marco Bove, Laura Bonzano

**Affiliations:** ^1^Department of Neuroscience, Rehabilitation, Ophthalmology, Genetics, Maternal and Child Health, University of Genoa, Genoa, Italy; ^2^Department of Experimental Medicine, Section of Human Physiology, University of Genoa, Genoa, Italy; ^3^Department of Developmental and Social Psychology, University of Padova, Padua, Italy; ^4^INSERM UMR1093-CAPS, Université Bourgogne Franche-Comté, UFR des Sciences du Sport, Dijon, France; ^5^Italian Multiple Sclerosis Foundation, Scientific Research Area, Genoa, Italy; ^6^IRCCS Ospedale Policlinico San Martino, Genoa, Italy

**Keywords:** functional near infrared spectroscopy, resting-state, functional connectivity, New-York Cognition Questionnaire, time-of-day

## Abstract

Time-of-day is rarely considered during experimental protocols investigating motor behavior and neural activity. The goal of this work was to investigate differences in functional cortical connectivity at rest linked to the time of the day using functional Near-Infrared Spectroscopy (fNIRS). Since resting-state brain is shown to be a succession of cognitive, emotional, perceptual, and motor processes that can be both conscious and nonconscious, we studied self-generated thought with the goal to help in understanding brain dynamics. We used the New-York Cognition Questionnaire (NYC-Q) for retrospective introspection to explore a possible relationship between the ongoing experience and the brain at resting-state to gather information about the overall ongoing experience of subjects. We found differences in resting-state functional connectivity in the inter-hemispheric parietal cortices, which was significantly greater in the morning than in the afternoon, whilst the intra-hemispheric fronto-parietal functional connectivity was significantly greater in the afternoon than in the morning. When we administered the NYC-Q we found that the score of the question 27 (“during RS acquisition my thoughts were like a television program or film”) was significantly greater in the afternoon with respect to the morning. High scores in question 27 point to a form of thought based on imagery. It is conceivable to think that the unique relationship found between NYC-Q question 27 and the fronto-parietal functional connectivity might be related to a mental imagery process during resting-state in the afternoon.

## 1. Introduction

Despite its potential impact, time-of-day is rarely considered during experimental protocols investigating motor and/or mental aspects of performance and the corresponding brain activity. Indeed, over the past two decades, some interesting insights came from the analysis of behavioral and neural activity during tasks, and from brain network parameters during resting-state. In particular, movement speed (Jasper et al., 2009), muscular force (Guette et al., 2005), and motor predictions of well-learned movements, such as walking, pointing, or writing (Gueugneau et al., 2009; Gueugneau and Papaxanthis, 2010; Debarnot et al., 2012), are modulated during the day. In this context, Bonzano et al. (2016) investigated task performance and the corresponding neural correlates with fMRI and showed a daily difference in both behavioral parameters and brain activation patterns, suggesting that motor performance is continuously updated throughout the day with a predominant role of the frontoparietal cortex and the cerebellum. Bonzano et al. (2015) also showed a relationship between the resting-state functional connectivity and a motor learning task performed immediately afterward. Finally, further insights from resting-state fMRI studies revealed differences in brain network parameters at different times of the day. Farahani et al. (2021) showed that during the evening functional topology is more efficient, and the somatomotor network contained the most highly connected areas in the brain. Regional Homogeneity (ReHo) was found to be greater in the morning compared to the evening in the occipital, parietal, and left temporal lobe, while greater ReHo was found in the evening in the frontal and anterior–posterior cingulate midline (Jiang et al., 2016). All this evidence suggests an impact of the time of the acquisition at both the behavioral and neural levels.

Here, we aimed to investigate differences in functional connectivity linked to the time of the day in cortical areas using functional Near-Infrared Spectroscopy (fNIRS; Niu et al., 2012, 2013), thus allowing the analysis of hemodynamic signals in ecological settings, e.g., while sitting in a quiet room, differently from fMRI which only allows investigations in horizontal body position in a noisy scanner. Further, resting-state brain is shown to be a succession of cognitive, emotional, perceptual, and motor processes that can be both conscious and nonconscious (Gonzalez-Castillo et al., 2021). Hence, studying self-generated thought can help in understanding brain dynamics. We used the New-York Cognition Questionnaire (NYC-Q; Gorgolewski et al., 2014) for retrospective introspection to explore a possible relationship between the ongoing experience and the brain at resting-state to gather information about the overall ongoing experience of subjects.

Horovitz et al. (2009) found that during deep sleep the posterior areas (bilateral inferior parietal cortices) strengthen their connectivity, whereas the connections between frontal and posterior regions are lost. Also, Sämann et al. (2011) showed that frontoparietal connections in default mode network are disrupted during deep sleep. Thus, following these findings, we hypothesized that parietal connections are stronger in the morning than in the afternoon, while frontoparietal connections are weaker in the morning compared to the afternoon.

## 2. Methods

### 2.1. Participants

Forty-one healthy volunteers were recruited (mean age = 29.7 years, SE = 1.5, 26 females) for the experiment, after giving their signed informed consent. None of them had a history of neurological disease. All the participants were naïve to the specific purpose of the study. The study was approved by the Regional Ethics Committee of Azienda Ospedaliera “San Martino,” Genoa, Italy (P.R. 271REG2017). The entire group was randomly divided into two sub-groups: the morning (AM) group, which included twenty participants (mean age = 31.9 years, SE = 2.6, 14 females) tested during the morning (from 9 a.m. to 12 a.m.); and the afternoon (PM) group, which included twenty-one participants (mean age = 27.7 years, SD = 1.4, 12 females) tested during the afternoon (from 2 p.m. to 5 p.m.). The choice of the time intervals was based on the literature, showing differences between behavioral parameters collected in a morning session (8–12 a.m.) and those acquired during an afternoon session (2–5 p.m.) (Gueugneau et al., 2015; Bonzano et al., 2016; Truong et al., 2022). To examine the gender proportion (male, female) between AM and PM groups, we used a Chi-square independence test. Moreover, we performed an unpaired t-test to evaluate differences in age between the two groups.

### 2.2. Experimental protocol

The experimental protocol consisted of a preliminary “dummy” task followed by 15 min of resting-state acquisition with fNIRS. At the end of the resting-state acquisition, participants had to answer the NYC-Q (Gorgolewski et al., 2014). During the experiment, participants wore the fNIRS cap. The montage was performed at the beginning and the average setup time was around 30 min. The fNIRS signal was registered only during the resting-state acquisition, and not during the dummy task or the NYC-Q.

On the basis of Albert et al. (2009) and Bonzano et al. (2015), before resting-state acquisition we proposed a dummy task, to direct conscious processing to the same task, ensuring that the resting period was not affected by different preceding activities. However, we did not collect responses during this task with the aim to measure cognitive abilities, but our goal was that participants maintained their attention focused on this task and not to others. Specifically, the dummy task lasted 4 min and required the participants to look at the computer screen. The computer displayed dynamic point-light of human whole-body movements or scrambled versions that showed the same individual dot motions but with random positions (Jastorff et al., 2006). These stimuli lasted 3 s each and were grouped into 30-s interleaved runs of 10 human and 10 scrambled motion stimuli. Participants had no active task to perform, but they had to watch the stimuli and try to discriminate between human and scrambled movements.

During fNIRS resting-state acquisition, participants were seated on a chair and instructed to stay still, close their eyes, and not focus their thoughts on anything in particular. The experiment was performed in a quiet and dimly lit room. The last part of the experiment consisted of answering the NYC-Q, to assess the relationship between intrinsic neural fluctuation and self-generated mental activity. The NYC-Q consists of 31 questions divided into two sections. The first section refers to the content of the self-generated thought (questions 1–23), while the second section refers to the form (questions 24–31). In detail, participants were asked to answer questions about the content and the form of the thoughts they had during the just finished experience (e.g., Q1: I thought about things I am currently worried about or Q25: My thoughts were in the form of words). For each question, they were asked to indicate how well each statement described their thoughts on a scale from 1 (“Completely did not describe my thoughts”) to 9 (“Completely did describe my thoughts”).

### 2.3. fNIRS signal acquisition and array configuration

fNIRS data were acquired with a continuous-wave, portable, multichannel NIRS system (NIRSport 2, NIRx Medical Technologies, Berlin, Germany), consisting of 16 LED illumination sources and 16 active detection sensors operating at two continuous wavelengths of near-infrared light (760 nm and 850 nm) to detect changes in concentration of oxy- (HbO) and deoxy- (HbR) hemoglobin. Optodes were arranged on a soft black tissue cap (EasyCap, Germany) on the participants’ heads to cover prefrontal, premotor, motor, sensory and parietal brain areas. The array was composed of 44 standard channels (3 cm) and 8 short-separation (SS) channels (8 mm). Table 1 lists standard channels, their MNI coordinates, and the associated Brodmann’s area (BA). The sampling frequency was set at 8.7 Hz.

**Table 1:** Description of the acquisition channels: optodes positions in the 10–10 system, MNI coordinates, cortical locations and Brodmann’s areas.

Ch	Label_S	Label_D	X	Y	Z	Hemisphere	Lobe	Cortical location	BA
1	CP1	C1	−27	−36	71	Left	Parietal	Postcentral gyrus	3
2	C3	C1	−42	−20	62	Left	Frontal	Precentral gyrus	4
3	FC1	FC3	−38	12	55	Left	Frontal	Middle frontal gyrus	6
4	FC1	FCz	−13	12	67	Left	Frontal	Superior frontal gyrus	6
5	FC1	C1	−26	−5	68	Left	Frontal	Superior frontal gyrus	6
6	C3	FC3	−50	−3	50	Left	Frontal	Precentral gyrus	6
7	Cz	C1	−17	−20	74	Left	Frontal	Precentral gyrus	6
8	CP1	P1	−24	−62	62	Left	Parietal	Superior parietal lobule	7
9	CP1	CPz	−16	−50	72	Left	Parietal	Postcentral gyrus	7
10	P3	P1	−32	−73	47	Left	Parietal	Superior parietal lobule	7
11	Pz	P1	−13	−73	56	Left	Parietal	Superior parietal lobule	7
12	FC1	F1	−23	26	56	Left	Frontal	Superior frontal gyrus	8
13	F3	F1	−31	39	41	Left	Frontal	Middle frontal gyrus	9
14	F3	FC3	−45	25	41	Left	Frontal	Middle frontal gyrus	9
15	Fz	F1	−9	41	50	Left	Frontal	Superior frontal gyrus	9
16	Fpz	Fp1	−12	67	0	Left	Frontal	Medial frontal gyrus	10
17	AF3	Fp1	−24	63	9	Left	Frontal	Middle frontal gyrus	10
18	AF3	AFz	−12	62	23	Left	Frontal	Superior frontal gyrus	10
19	AF3	F1	−23	52	32	Left	Frontal	Superior frontal gyrus	10
20	P3	CP3	−46	−61	46	Left	Parietal	Inferior parietal lobule	39
21	C3	CP3	−52	−34	52	Left	Parietal	Postcentral gyrus	40
22	CP1	CP3	−39	−48	60	Left	Parietal	Inferior parietal lobule	40
23	CP2	C2	27	−35	71	Right	Parietal	Postcentral gyrus	3
24	C4	C2	42	−21	62	Right	Frontal	Precentral gyrus	4
25	Cz	C2	17	−21	75	Right	Frontal	Precentral gyrus	6
26	FC2	FCz	14	13	66	Right	Frontal	Superior frontal gyrus	6
27	FC2	FC4	39	12	54	Right	Frontal	Middle frontal gyrus	6
28	FC2	C2	27	−4	68	Right	Frontal	Superior frontal gyrus	6
29	C4	FC4	52	−4	48	Right	Frontal	Precentral gyrus	6
30	Pz	P2	15	−73	57	Right	Parietal	Superior parietal lobule	7
31	P4	P2	33	−74	48	Right	Parietal	Superior parietal lobule	7
32	CP2	CPz	17	−50	73	Right	Parietal	Postcentral gyrus	7
33	CP2	P2	25	−62	63	Right	Parietal	Superior parietal lobule	7
34	FC2	F2	24	26	55	Right	Frontal	Superior frontal gyrus	8
35	Fz	F2	10	41	50	Right	Frontal	Superior frontal gyrus	9
36	F4	F2	30	40	41	Right	Frontal	Middle frontal gyrus	9
37	F4	FC4	44	25	40	Right	Frontal	Middle frontal gyrus	9
38	Fpz	Fp2	13	67	0	Right	Frontal	Medial frontal gyrus	10
39	AF4	AFz	13	61	24	Right	Frontal	Superior frontal gyrus	10
40	AF4	Fp2	25	63	9	Right	Frontal	Middle frontal gyrus	10
41	AF4	F2	22	52	33	Right	Frontal	Superior frontal gyrus	10
42	P4	CP4	46	−62	47	Right	Parietal	Inferior parietal lobule	39
43	CP2	CP4	39	−49	60	Right	Parietal	Postcentral gyrus	40
44	C4	CP4	52	−35	52	Right	Parietal	Postcentral gyrus	40

S, source; D, detector.

### 2.4. Resting-state data pre-processing and statistical analysis

Data pre-processing and analyses were performed in MATLAB (MathWorks, MA, United States) through in-house scripts utilizing Homer3 functions. First, noisy channels were removed, and the remaining channels were converted in changes of optical density. Motion artifacts were identified as the segment of data around time points that exhibited a signal change greater than almost one of two imposed thresholds. One threshold was the standard deviation threshold which was computed as 12 times the standard deviation of the entire channel signal, the other was the amplitude threshold and was set to 0.5 OD. Then, the signal was band-pass (0.009–0.08 Hz; Mesquita et al., 2010; Niu et al., 2012). The differential path-length factor was computed considering the age of the participant (Scholkmann and Wolf, 2013), then optical density data were converted in concentration changes, and the SS channels were regressed out. In the end, temporal frames that corresponded to identified motion artifacts in almost one channel were discarded. Then, concentration change signals of channels belonging to the same hemisphere and BA were averaged obtaining 18 regions of interest (ROIs; BA10, BA9, BA8, BA7, BA6, BA4, BA3, BA39, and BA40 for the left and right hemispheres). The first minute of the acquisition was discarded to use only the stable signal (Lu et al., 2010; Cai et al., 2018), thus the following steps were performed on 14 min of acquisition. For each participant, the individual correlation matrix was obtained by calculating Pearson correlation between each couple of ROIs resulting in an 18 × 18 *r*-values matrix. Only *r*-values, reflecting the strength of functional connectivity (FC) between two correlated ROIs, associated with *p* < 0.05 were considered significant and successively considered. After calculating participants’ correlation matrixes, three group correlation matrices were obtained as the average of the individual correlation matrices of the participants included in the corresponding group: the total group correlation matrix, the AM correlation matrix, and the PM correlation matrix. All pairs of ROIs that had an *r*-value <0.2 (Cai et al., 2018, 2019; Wang et al., 2020) were considered as not functionally connected. Finally, to assess the statistical difference between the AM and the PM correlation matrix, individual correlation matrices were Z-transformed and then each box of the matrix was compared between the two groups through a non-parametric test (Wilcoxon rank sum test, *p* < 0.05). A statistical difference for one box means different FC in the morning compared to the afternoon between the ROIs linked to that box.

### 2.5. NYC-Q analysis

Here, we were specifically interested in the form of thought; therefore, unpaired t-test was used to assess whether there was a statistical difference between the AM and PM groups in responses to the single questions belonging to the second section of the questionnaire. Then, to explore a possible relationship between the form of thoughts and cortical functional connectivity, we performed the Pearson correlation between score and FC, taking into account the questions and connections that were found to be statistically different between the morning and the afternoon sessions and applying Bonferroni correction.

## 3. Results

### 3.1. Demographic characteristics

The Chi-square independence test showed that gender proportions did not differ significantly between the AM and the PM groups (*X*^2^ = 0.73; df = 1; *p* = 0.39). The unpaired t-test showed no significant differences in age between groups (*p* = 0.15).

### 3.2. fNIRS region-to-region functional connectivity

**Figure 1:**
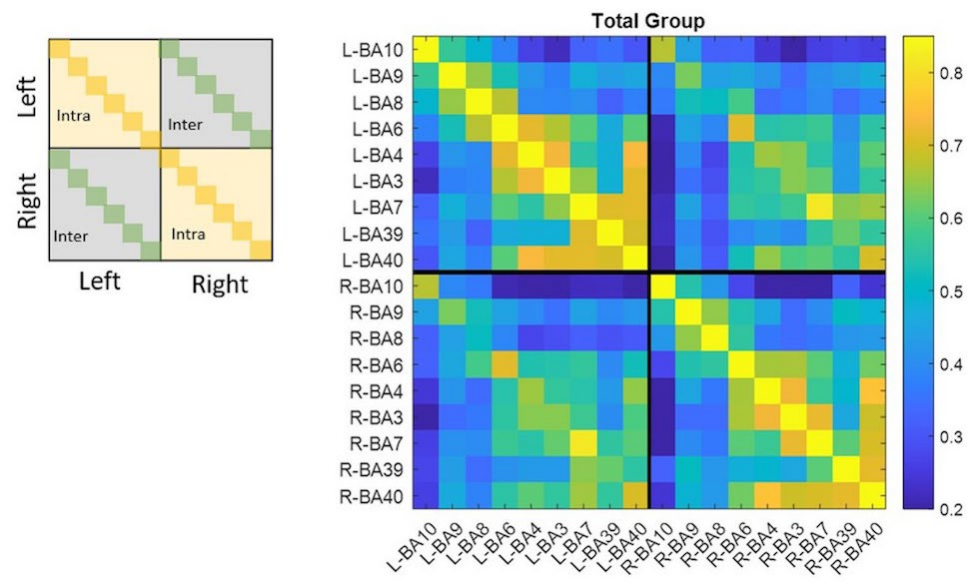
Matrixes were organized in four dials to divide the left hemisphere from the right one and recognize inter- and intra-hemispheric functional connections. Diagonals correspond to homologous areas in the dials which contain inter-hemispheric correlations, while diagonals in the other dials had correlations equal to one because they represent autocorrelations. All values ≤0.2 are dark blue and identify pair of ROIs that resulted not functionally connected. All the other values follow colormap legend.

Figure 1 shows the correlation matrix calculated for the whole group. Pairs of ROIs that resulted not being functionally connected are represented through dark blue boxes (*r* < 0.02). The total group correlation matrix, among intra-hemispheric connections, showed two clusters of highly functionally connected regions including: (i) BA10, BA9, BA8, and BA6; (ii) BA6, BA4, BA3, BA7, BA39, and BA40. In the second cluster, the weakest connections were between BA39 and BA6 (left *r*_BA39-BA6_ = 0.46; right *r*_BA39-BA6_ = 0.48), BA4 (left *r*_BA39-BA4_ = 0.48; right *r*_BA39-BA4_ = 0.49), and BA3 (left *r*_BA39-BA3_ = 0.48; right *r*_BA39-BA3_ = 0.46). Outside these two clusters, the highest correlations were between left BA7 and BA9 (*r*_BA7-BA9_ = 0.47) and between right BA9 and both BA39 (*r*_BA9-BA39_ = 0.51) and BA40 (*r*_BA9-BA40_ = 0.49), whereas the weakest connections were between left BA10 and left BA3 (*r*_BA10-BA3_ = 0.22) and right BA10 and right BA40 (*r*_BA10-BA40_ = 0.24). Among inter-hemispheric functional connections, the highest correlations were between homologous areas (*r*_BA10_ = 0.67, *r*_BA9_ = 0.62, *r*_BA8_ = 0.52, *r*_BA6_ = 0.71, *r*_BA4_ = 0.65, *r*_BA3_ = 0.64, *r*_BA7_ = 0.82, *r*_BA39_ = 0.62, *r*_BA40_ = 0.70), among BA6, BA4, BA3, and BA7, and between BA40 and BA39 (*r*_BA40-BA39_ = 0.56), BA7(*r*_BA40-BA7_ = 0.66), BA3(*r*_BA40-BA3_ = 0.56), BA4 (*r*_BA40-BA4_ = 0.61), and BA6 (*r*_BA40-BA6_ = 0.55). The AM group correlation matrix had the same two main clusters in both the left and right hemispheres (intra-hemispheric connections). Outside these clusters, the highest correlations in addition to those found in the total group were between left BA9 and left BA39 (*r*_BA9-BA39_ = 0.46). ROIs with the weakest connections in the whole group here were considered not connected, while left BA10 with left BA7 (*r*_BA10-BA7_ = 0.25) and right BA10 with right BA6 (*r*_BA10-BA6_ = 0.23) were the weakest connections. The highest inter-hemispheric connections were the same as the total group. Finally, the PM group correlation matrix, besides showing the same previous pattern in both intra-hemispheres, had also a high functional connection between left BA9 and left BA40 (*r*_BA9-BA40_ = 0.48). The weakest connections were different from the total group, they were between the right BA10 and both right BA4 (*r*_BA10-BA4_ = 0.22) and right BA7 (*r*_BA10-BA7_ = 0.22). The highest inter-hemispheric connections were the same as the total group.

**Figure 2:**
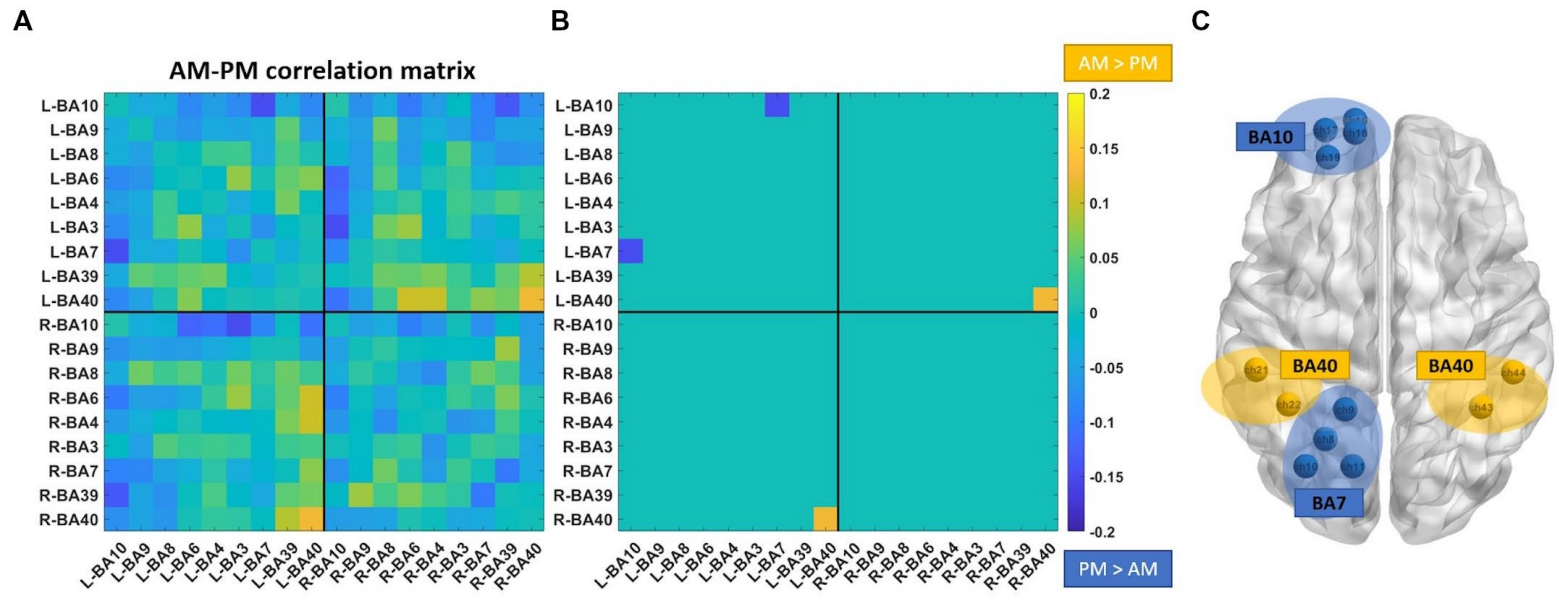
Comparison of the correlation matrixes between the morning and the afternoon groups. **(A)** Difference between AM correlation matrix and PM correlation matrix. **(B)** Statistically significant differences between the two correlation matrixes. **(C)** Brain visualization of statistically significant differences. Channels belonging to left and right BA40, left BA10 and left BA7 are shown.

The difference between the AM and PM correlation matrices is reported in Figure 2. Only two functional connections survived the statistical comparison: the connection between left BA40 and right BA40 that is greater in the morning than in the afternoon (*p* = 0.038); the connection between left BA7 and left BA10 that was greater in the afternoon (*p* = 0.036) than in the morning.

### 3.3. Thoughts characterization and relationships with functional connectivity

**Figure 3:**
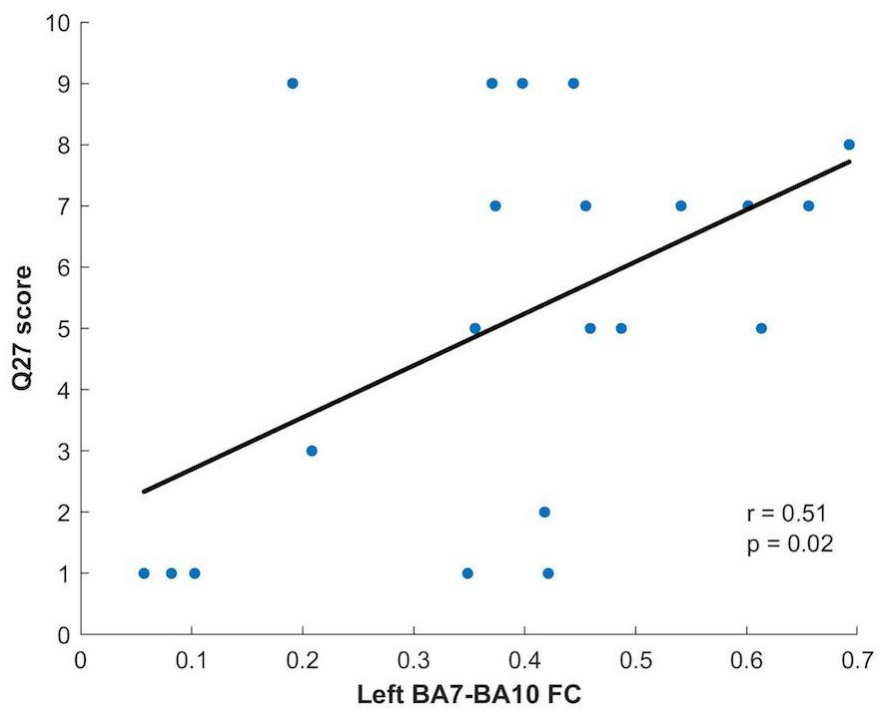
Relationship between Q27 score and Left BA7-BA10 FC for the PM group.

Concerning the NYC-Q, a significant difference between AM and PM groups was found for question 27 (“during RS acquisition my thoughts were like a television program or film”) and question 29 (“during RS acquisition my thoughts had a clear sense of purpose”). Question 27 showed significantly greater scores in the afternoon compared to the morning (*p* = 0.028), while question 29 showed significantly greater values in the morning compared to the afternoon (*p* = 0.041). The scores of these questions found to be significantly different between groups were correlated, separately for each group, with the FC of the connections that were shown to be different in the morning compared to the afternoon: left BA7 - left BA10 and left BA40 - right BA40. A significant correlation, surviving Bonferroni correction (*p* = 0.025), was found in the afternoon group between the question 27 score and the left BA7-left BA10 FC (*p* = 0.019; Figure 3).

## 4. Discussion

In this work, the main findings we found were that the functional connectivity in the morning between inter-hemispheric parietal cortices (left BA40 and right BA40) was significantly greater than that evaluated in the afternoon. This may indicate an update of internal predictive models early in the morning, after sleep, to prepare the organism for the upcoming day, as the parietal cortex is concerned with high-level prediction, such as strategic planning actions (Blakemore and Sirigu, 2003; Sirigu et al., 2016). An internal forward model is a neural network that mimics the causal flow of the physical process by predicting the future sensorimotor state (e.g., position, velocity) given the goal of the movement, the efferent copy of the motor command, and the current state (Wolpert and Flanagan, 2001). During movement execution, predictions are compared with sensory feedback from the periphery. Any discrepancy constitutes an error signal that can update the internal forward model (i.e., better predictions) and the controller (i.e., better performance) via a “self-supervised process.”

On the other hand, the intra-hemispheric fronto-parietal functional connectivity between left BA7 and left BA10 was significantly greater in the afternoon than in the morning. Further, when we administered the NYC-Q we found that the score of the question 27 (“during RS acquisition my thoughts were like a television program or film”) was significantly greater in the afternoon with respect to the morning. High scores in question 27 point to a form of thought based on an imagery dimension (Gorgolewski et al., 2014). Interestingly, this score significantly correlated only with the functional connectivity between left BA7 and left BA10 in the afternoon, and it is conceivable to think that this correlation might be related to mental imagery. Indeed, previous studies have shown the activation of both regions during mental movements (Hardwick et al., 2018).

Left BA7 seems to be a core region for visual mental imagery: in an fMRI study contrasting visual stimulation and visual imagery, the results have shown that for most of the participants activity in left BA7 during visual imagery was significantly larger than that observed during image perception (Knauff et al., 2000). Another result consistent with this idea comes from an fMRI study on music imagery and real performance in pianists (Meister et al., 2004): the hemodynamic pattern in left BA7 observed during action execution was indistinguishable from the one observed during music imagery. Unlike BA7, BA10 is not classically associated with visual imagery; on the other hand, it seems crucially related to the processing of internal representations. BA10 activity has found to be correlated with the amount of intentional effort involved in self-awareness and the imagination of future events (Goldberg et al., 2006), and it seems to be involved in the evaluation of internally generated information (Christoff and Gabriel, 2000; Turner et al., 2008). Taken together, these pieces of information suggest that the two regions interact to generate and monitor a vivid experience of mental imagery. Overall, the results confirm the potential usefulness of retrospective introspection as a viable mean to explore the relationship between the ongoing subjective experience of participants and the key characteristics of their brain during resting state.

In conclusion, we suggest that the observed cortical hemodynamic changes at rest during the day are not only related to functional connectivity changes in cortical networks but can reflect a different modality of thoughts during resting-state condition. We firmly believe that resting-state functional connectivity studies should be coupled with questionnaires more often (such as the NYC-Q). It is worth noting that the information provided by the questionnaires alone might have important practical implications: the responses collected might be used as a broad guide to choose the best time when to perform a specific activity (e.g., in the present study participants were more prone to vivid mental imagery in the afternoon). However, using questionnaires in resting-state functional connectivity studies might be even more enlightening: for instance, the present findings might entice to non-invasively stimulate (e.g., with random noise stimulation left BA7 and left BA10 in order to enhance mental imagery). Furthermore, as highlighted in the present work, adding questionnaires in resting-state functional connectivity studies might yield two important advantages: on one hand, it might significantly enrich the current knowledge on resting-state functional connectivity; secondly, it might provide a way to account for the interindividual variability which is observed in resting-state functional connectivity studies (especially for the variability created by different subjective experience).

Finally, to strengthen the results of this study, we could suggest to overcome the limitations of the present work concerning the experimental protocol, which included two different groups of participants. Further studies should test the same group of healthy volunteers in both the morning and afternoon sessions. In addition, more testing times during the day could be considered.

## Data availability statement

The original contributions presented in the study are included in the article/supplementary material, further inquiries can be directed to the corresponding author/s.

## Ethics statement

The studies involving human participants were reviewed and approved by Regional Ethics Committee of Azienda Ospedaliera “San Martino,” Genoa, Italy (P.R. 271REG2017). The patients/participants provided their written informed consent to participate in this study.

## Author contributions

CI, MBo, and LB gave substantial contributions to the conception and design of the work. CI and MBi did the data acquisition. CI, SB, SC, and LB performed the analysis and interpretated the data. MBo, MBi, and LB drafted the work. CP and GB revised it critically for important intellectual content. All authors contributed to the article and approved the submitted version.

## Conflict of interest

The authors declare that the research was conducted in the absence of any commercial or financial relationships that could be construed as a potential conflict of interest.

## Publisher’s note

All claims expressed in this article are solely those of the authors and do not necessarily represent those of their affiliated organizations, or those of the publisher, the editors and the reviewers. Any product that may be evaluated in this article, or claim that may be made by its manufacturer, is not guaranteed or endorsed by the publisher.
